# Understanding Methods for Estimating HIV-Associated Maternal Mortality

**DOI:** 10.1155/2012/958262

**Published:** 2011-09-28

**Authors:** James E. Rosen, Isabelle de Zoysa, Karl Dehne, Viviana Mangiaterra, Quarraisha Abdool-Karim

**Affiliations:** ^1^Futures Institute, New London Turnpike, Glastonbury, CT 06033, USA; ^2^Lungarno Guicciardini, 17, 50125 Firenze, Italy; ^3^UNAIDS, Avenue Appia, Geneva 1211, Switzerland; ^4^World Health Organization, Avenue Appia, Geneva 1211, Switzerland; ^5^CAPRISA, Nelson R Mandela School of Medicine, University of Kwazulu-Natal, Congella 4013, South Africa

## Abstract

The impact of HIV on maternal mortality and more broadly on the health of women, remains poorly documented and understood. Two recent reports attempt to address the conceptual and methodological challenges that arise in estimating HIV-related maternal mortality and trends. This paper presents and compares the methods and discusses how they affect estimates at global and regional levels. Country examples of likely patterns of mortality among women of reproductive age are provided to illustrate the critical interactions between HIV and complications of pregnancy in high-HIV-burden countries. The implications for collaboration between HIV and reproductive health programmes are discussed, in support of accelerated action to reach the Millennium Development Goals and improve the health of women.

## 1. Introduction

While recent reports indicate declining trends in maternal mortality [1, 2], at the current rate of progress, most countries remain unlikely to reach the Millennium Development Goal 5 and its target of reducing the maternal mortality ratio (MMR) by 75% between 1990 and 2015. The adverse effect of HIV on women's health in sub-Saharan Africa appears to be an important reason for poor progress [1, 3].

The contribution of HIV to maternal mortality has been recognized for over a decade [4, 5], but it remains poorly documented and understood. The number and proportion of maternal deaths associated with HIV have been difficult to determine with precision because of various conceptual and measurement challenges [1, 2]. This information is critical to plan services for women in need, including HIV-infected pregnant women.

This paper aims to enhance the understanding of the methods used to estimate HIV-associated maternal deaths and how they affect global, regional, and country estimates. Country examples of likely patterns of mortality among women of reproductive age are provided to illustrate the critical interactions between HIV and maternal mortality in high-HIV-burden countries.

## 2. Methods for Estimating HIV-Associated Maternal Deaths

### 2.1. Definitions

In the International Statistical Classification of Diseases and Related Health Problems, Tenth Revision, 1992 (ICD-10), WHO defines maternal death as the death of a woman while pregnant or within 42 days of termination of pregnancy, irrespective of the duration and site of the pregnancy, from any cause related to or aggravated by the pregnancy or its management but not from accidental or incidental causes [6]. Maternal deaths are either *direct* obstetric deaths resulting from obstetric complications of the pregnant state (pregnancy, delivery, and postpartum), from interventions, omissions, incorrect treatment, or from a chain of events resulting from any of the above, or *indirect *deaths resulting from previous existing disease or disease that developed during pregnancy, such as anaemia and malaria, which was aggravated by physiological effects of pregnancy. In some instances, deaths during pregnancy or the postpartum period might be due to causes that are incidental to pregnancy, such as accidents and violence. These are defined in ICD as pregnancy-related deaths, but are not included as part of maternal deaths. Pregnancy-related deaths comprise maternal deaths due to direct and indirect causes and also deaths due to accidental or incidental events that occur when a woman is pregnant. 

Some of the deaths of pregnant women with HIV are incidental deaths due to an AIDS-defining condition, without any obvious association with pregnancy. These deaths are therefore classified as pregnancy-related deaths, but not maternal deaths. In practice, the distinction between incidental and indirect causes of death is difficult to make, especially in the case of HIV. The effect of pregnancy on HIV disease progression is uncertain [7–9], but HIV would not likely be an indirect cause of death except in advanced stages of the disease. It is also not clear to what extent HIV infection has an effect on the maternal outcomes of pregnancy [7, 8]. However, there is some evidence that HIV can aggravate obstetric conditions that can lead to death, such as sepsis, haemorrhage, and septic abortion [9, 10].

**Figure 1:**
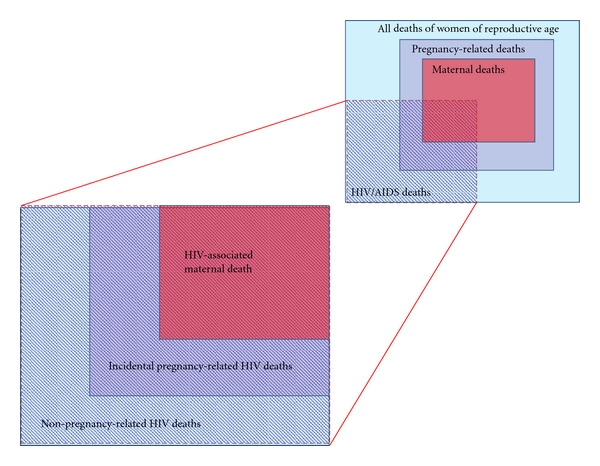
The intersecting epidemics of HIV and maternal mortality.

Figure 1 is a graphical representation of the intersection of HIV deaths and maternal deaths among all deaths of women of reproductive age (15–49 years). In this paper, we use the term “HIV-associated maternal death” to describe the death of a pregnant woman with HIV in which the HIV infection was present at the time of a death from direct obstetric causes or else was an indirect cause of maternal death. The figure shows that HIV-associated maternal deaths are a subset of what we call “pregnancy-related HIV deaths,” which include all deaths in HIV-positive women that take place during pregnancy, childbirth, and 42 days postpartum. These include deaths from HIV disease that were incidental to the pregnancy. This figure does not attempt to show the relative distribution of deaths in the various categories. For orders of magnitude, it is useful to recall here that HIV/AIDS and complications of pregnancy and childbirth are the two leading causes of death in young adult women globally, accounting for 19% and 15% of all deaths in women aged 15–44 years, respectively [11].

### 2.2. Measurement Challenges

In the real world, identifying maternal deaths and determining their causes, in order to differentiate deaths due to direct or indirect causes, or to incidental causes, poses many challenges. Most developing countries do not have civil registration systems to record the death of a woman of reproductive age, and identification of maternal deaths usually requires special methods of investigation, such as household surveys. In addition, there are well-documented problems with regard to the identification of cause of death in general and maternal death in particular, which are exacerbated in the context of HIV [12–14]. Ideally, HIV-associated maternal deaths should be counted using the ICD-10 classification system, along with other causes of maternal deaths—direct and indirect. In practice, this is challenging, due to insufficient or inaccurate information on the circumstances of death and limited capacities to correctly use standards to assign and code causes of death. In particular, information about the woman's pregnancy status or HIV status is often missing or withheld. Even when deaths are medically certified, applying the ICD rules when deaths are associated with multiple comorbidities is not straightforward [2, 10]. With ICD-10, WHO recommends the inclusion of a checkbox on the death certificate for recording a woman's pregnancy status at the time of death, but this has not been implemented in many countries, and therefore some AIDS deaths in pregnant women are not identified as pregnancy-related. The overall result is that most countries report incomplete and inaccurate data on numbers and causes of maternal and pregnancy-related deaths, and the precise contribution of HIV is poorly documented.

### 2.3. Methods of Estimating HIV-Associated Maternal Deaths

In the run-up to the 2015 deadline to achieve the Millennium Development Goals, increased efforts are being made to track progress in reducing maternal mortality. In 2010, two major reports provided estimates of maternal mortality trends at global, regional, and country levels [1, 2]. Maternal mortality estimates were based on data collected through a variety of methods, including civil registration systems, household surveys, special studies, censuses, and verbal autopsies [2]. Different statistical modeling approaches were used to estimate the contribution of HIV to maternal mortality.

Hogan and colleagues, at the Institute for Health Metrics and Evaluation (IHME) and other academic institutions, estimated levels and trends in maternal mortality for 181 countries between 1980 and 2008 and assessed the impact of HIV on these estimates. Details on the IHME methodological approach can be found in the published report [1], webannex [15], and model output data published on the IHME website (http://www.who.int/gho/maternal_health/mortality/maternal/en/index.html). In summary, IHME's multivariate model included HIV prevalence as one of several covariates to estimate total maternal deaths, including what the authors call “HIV-related maternal deaths.” To distinguish the HIV-related deaths among the total number of maternal deaths, IHME conducted a counterfactual analysis that explored the effect on maternal mortality of reducing HIV prevalence to zero, by setting the HIV covariates of the estimated model to zero values. The difference between the two outputs produced the number of HIV-related maternal deaths. The primary source of data used in many countries was surveys or censuses, which do not exclude incidental deaths in pregnant women. Therefore, the numbers reported by IHME in those countries relate to our definition of pregnancy-related HIV death, including both HIV-associated maternal deaths and HIV deaths that are incidental to the pregnancy. Hogan and colleagues indicate that this approach would tend to bias the maternal mortality estimates upward, but note that there is no evidence-based analytical strategy to identify the proportion of pregnancy-related deaths that are incidental. 

The UN-affiliated Maternal Mortality Estimation Inter-Agency Group (MMEIG), together with academics from the University of California at Berkeley, developed estimates of indirect maternal deaths due to HIV as part of a larger effort to estimate country, regional, and global maternal mortality levels and trends between 1990 and 2008 [2, 16]. The interagency group also took available data on total deaths in women of reproductive age as the starting point, but applied different statistical models and assumptions to generate estimates of maternal deaths. In the first instance, only maternal, non-HIV-related deaths were computed, by subtracting the fraction of estimated HIV deaths in the pregnancy-related period (which corresponds to our definition of pregnancy-related HIV deaths). Then the number of indirect maternal deaths due to HIV was computed and added back to obtain the total number of estimated maternal deaths. 

The steps in this process were as follows (see also [2], data files available on the WHO web site http://www.who.int/gho/maternal_health/mortality/maternal/en/index.html). First, a statistical model estimated the fraction of HIV deaths in women of reproductive age occurring during pregnancy or within 42 days of termination of pregnancy. The calculations required assumptions about the relative risk of dying from HIV of a pregnant versus a nonpregnant woman (reflecting both the decreased fertility of HIV-positive women and the increased mortality risk of HIV-positive pregnant women). This latter parameter was developed through a combination of expert opinion and tests of model fitness to reach a value of 0.4, which was applied to all countries. These deaths were removed from the computation of maternal deaths due to direct obstetric causes or to indirect causes other than HIV. Then a constant fraction of these deaths, counted as indirect maternal deaths, was added back to calculate total maternal deaths. For the estimation of these HIV-related indirect maternal deaths, it was assumed that half of the total pregnancy-related deaths among HIV-infected women were indirect maternal deaths, the other half being incidental to the pregnancy. This value was chosen to minimize distribution errors given the scarcity of appropriate data for estimating correct proportion of HIV deaths that are incidental (which can vary over time and place).

## 3. Results Based on the Different Methods

### 3.1. Worldwide HIV-Associated Maternal Deaths

The interagency group estimated 21,000 HIV-associated maternal deaths (uncertainty interval 15,341–29,120), or 6% of the worldwide total of 358,000 maternal deaths in 2008. For the same year, the IHME calculated that 61,400 fewer maternal deaths (18%) would have occurred in the absence of HIV (uncertainty interval 58,200–66,400), of an estimated total of 342,900 maternal deaths. As noted above, the interagency model computed pregnancy-related HIV deaths and assumed that half of those were indirect maternal deaths. Direct maternal deaths in which HIV was present, and possibly a contributing factor, were not counted. The other model computed primarily pregnancy-related HIV deaths. Should the IHME model have used the interagency assumption about the proportion of pregnancy-related HIV deaths that are truly maternal, their estimated numbers of HIV-associated maternal death and total number of maternal deaths would have been reduced accordingly.

### 3.2. Regional Variation in HIV-Associated Maternal Deaths

Both models show that the vast majority of HIV-associated maternal deaths occur in sub-Saharan Africa (86% in the interagency model and 92% in the IHME model) (Table 1). As a fraction of all maternal deaths, HIV-associated maternal deaths are also highest in the sub-Saharan region (9% under the interagency model and 32% in the IHME model). In other regions, HIV-associated maternal deaths account for 6% or less of all maternal deaths.

### 3.3. HIV-Associated Maternal Deaths by Country

The ranking of countries with the highest number of HIV-related maternal deaths varies substantially by model (Table 2). Nigeria is consistently ranked first, although the estimated number of deaths per year ranges widely, between 2,472 for the interagency model and 10,422 for the IHME model. Six countries make the top ten under both model estimates—Kenya, Malawi, Mozambique, Nigeria, Tanzania, and Uganda. However, their ranking differs under each model. Only one country outside sub-Saharan Africa, India, is in the top ten.

The countries with the highest fraction of HIV-related maternal deaths are shown in Table 3. The list is dominated by countries in Southern Africa that have been hardest hit by the HIV epidemic. In many of these countries, HIV-associated maternal deaths represent a substantial proportion of total maternal deaths. Both the interagency and IHME approaches estimate the fraction at greater than 70% for Botswana and Swaziland and over one-third for all countries in the top ten.

### 3.4. Relative Impact of HIV on Pregnancy-Related and Maternal Mortality at Country Level

**Figure 2:**
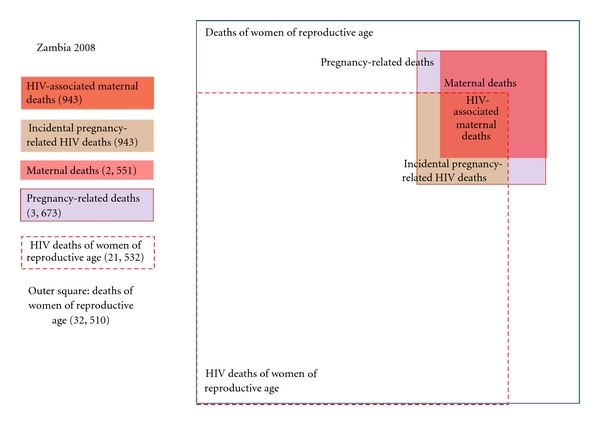
Intersection of HIV and maternal mortality in Zambia, 2008. Source: http://www.who.int/gho/maternal_health/mortality/maternal/en/index.html.

Figure 2 shows the substantial impact HIV has on the health of women in Zambia, a high-prevalence country. Based on the interagency group estimates, 21,532 or two-thirds of the 32,510 deaths of women of reproductive age in 2008 can be attributed to HIV. Of the total of 3,673 pregnancy-related deaths, 2,551 are maternal deaths. Of maternal deaths, 943 (37%) are estimated to be HIV associated. Another 943 women are estimated to die from incidental HIV deaths during pregnancy. 

**Figure 3:**
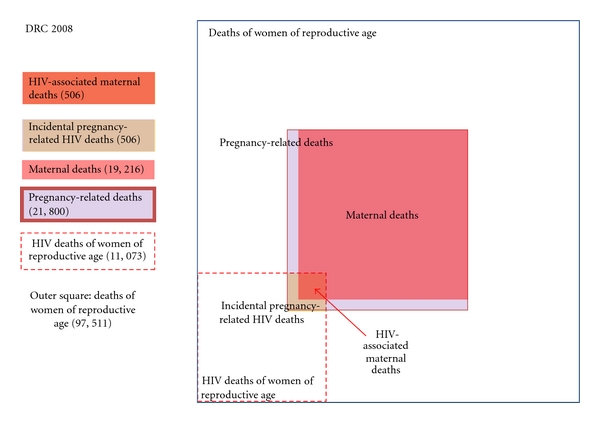
Intersection of HIV and maternal mortality in DRC, 2008. Source: http://www.who.int/gho/maternal_health/mortality/maternal/en/index.html.

**Figure 4:**
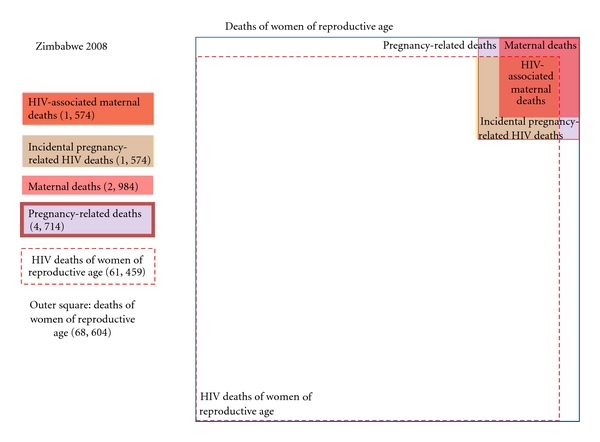
Intersection of HIV and maternal mortality in Zimbabwe, 2008. Source: http://www.who.int/gho/maternal_health/mortality/maternal/en/index.html.

Figure 3 shows the pattern in the Democratic Republic of Congo. In that country, a smaller proportion of deaths among women of reproductive age are reported to be associated with HIV, and a larger proportion of deaths are due to complications of pregnancy and childbirth. The opposite would seem to be the case in Zimbabwe, where the overwhelming majority of deaths among women of reproductive age are associated with HIV, as shown in Figure 4. Similar charts can be produced to support more detailed assessments of the situation in other affected countries.

## 4. Comparison of Methods and Results

We examined some of the challenges in defining and counting HIV-associated maternal deaths and reviewed two recent methodological approaches for their estimation. (This paper does not attempt to explain why the interagency and IHME estimates for maternal deaths might differ. For further discussion, see Section 3.6 of [2].) Both approaches address the well-known conceptual and practical difficulties in assessing a pregnancy-related death in the presence of HIV. They use statistical models to estimate the impact of HIV on levels and trends of maternal mortality, combining robust data sources with assumptions regarding key parameters about which little information exists. The interagency group estimates of indirect maternal deaths due to HIV rest on assumptions about the relative risk of HIV mortality in pregnant versus nonpregnant women and the proportion of pregnancy-related HIV mortality that can be considered maternal. IHME uses a counterfactual method that compares results with and without the HIV epidemic. 

There are major differences in the way each model identifies pregnancy-related HIV deaths and examines the association of HIV with maternal deaths. The IHME model produces somewhat higher results, with the number of HIV-associated maternal deaths (61,400) about three times higher than the interagency estimates. In part because the IHME model has a lower overall number of maternal deaths, the fraction of maternal deaths associated with HIV is substantially higher when compared to the interagency model. Regional estimates of both numbers and affected fraction follow a similar pattern between models. At the country level, the models produce similar rankings in terms of the most affected countries, although the estimated numbers and affected fractions vary. 

These comparisons should be made with caution, partly because of the different model approaches and in how the results are presented. The interagency estimate distinguishes between incidental pregnancy-related HIV deaths and indirect HIV-related maternal deaths, to keep strictly to the ICD-10 definition of maternal death, without being able to fully delineate the differences between the two. The IHME model notes that the evidence to identify the proportion of pregnancy-related deaths that are incidental is scant, and its estimates are based on total numbers of pregnancy-related deaths in which HIV was present, while recognizing that this strategy biases the estimates upwards. 

Despite these differences, estimates using the different methods clearly reinforce that the HIV epidemic is having a profound influence on maternal mortality and, more broadly, pregnancy-related mortality. Worldwide estimates of the fraction of maternal deaths due to HIV range between 6 and 18%, with the majority of HIV-associated maternal deaths in the sub-Saharan region, where the affected fraction is between 9–32%. Country rankings between models are similar and provide a general but not precise estimate of the HIV epidemic's contribution to maternal mortality. 

More detailed examination at country level of the impact of HIV on maternal and pregnancy-related deaths shows diverse patterns. National estimates are usually based on few data points and are subject to all the caveats mentioned earlier about the identification of pregnancy-related deaths and the classification of cause of death. Nonetheless, they give an idea of the severe adverse effect of the HIV epidemic on women's health in high-burden countries.

## 5. Conclusions

Despite methodological challenges, assessments of trends in maternal mortality clearly indicate that HIV has become a leading cause of death during pregnancy and the postpartum period in countries with high HIV prevalence and that the HIV epidemic has slowed down progress in improving maternal health.

Improved knowledge of the contribution of HIV to maternal and pregnancy-related mortality should help to direct scarce resources to appropriate policy and programmatic responses and spur better collaboration between HIV and reproductive health services [1, 17]. We hope that our examination of different approaches used to assess the interactions between HIV and maternal mortality will stimulate improvements in systems to document maternal deaths and assess their causes. 

It would be useful to distinguish between maternal HIV-associated death due to direct obstetric causes or indirect HIV-related causes and incidental pregnancy-related HIV deaths. The new ICD-10 amendment introducing a new code for an “indirect maternal death” is a step forward. However, this is likely to continue to remain a source of misclassification, given the ongoing clinical uncertainty around the relationship between HIV and other causes of maternal death. Further discussion among experts in ICD-compliant death certification and coding is needed to help clarify how best such interrelated causes of deaths should be classified. Adoption of a common terminology among researchers and health service providers would be a further benefit. 

There is also an urgent need to carry out systematic reviews of existing studies on the key parameters that underpin the estimates. These include studies on the relative risk of maternal death in HIV-infected versus uninfected pregnant women and the relative risk of dying from HIV disease of a pregnant versus a nonpregnant woman. More studies are also needed to elucidate the mechanisms by which HIV can contribute to direct or indirect maternal deaths. 

Nonetheless, given the close, almost inextricable interactions between HIV and other causes of death during and around pregnancy and inadequate systems for civil registration in most countries, it will remain difficult to quantify the precise impact of HIV on maternal mortality in the foreseeable future. This should not serve as an excuse for inaction. Urgent measures are required to provide the many girls and young women who face the dual risks of HIV infection and pregnancy with the services that they need. In settings with a high HIV burden and continuing high maternal mortality ratios, especially in sub-Saharan Africa, this means scaling up comprehensive and integrated programmes that include *both* improved HIV treatment and care and improved reproductive health services [9].

## Figures and Tables

**Table 1:** HIV-associated maternal deaths by region, 2008, by model.

MDG region	Number of HIV-associated maternal deaths		Proportion of all maternal deaths	
	Interagency	IHME	Interagency	IHME
World total	21,000	61,436	5.8%	17.9%
Developed regions	90	45	5.6%	3.4%
CIS countries	70	35	4.7%	2.6%
Developing regions	21,000	61,356	5.8%	18.0%
Africa	18,000	56,446	8.9%	31.5%
Northern Africa	10	28	0.3%	1.2%
Sub-Saharan Africa	18,000	56,418	9.0%	31.9%
Asia	1,700	4,358	1.2%	2.9%
Eastern Asia	80	42	1.0%	0.5%
South Asia	1,300	3,632	1.2%	3.0%
South-Eastern Asia	310	682	1.7%	3.9%
Western Asia	—	2	0.0%	0.0%
Latin America and the Caribbean	480	510	5.2%	6.4%
Oceania	10	42	1.1%	5.8%

**Table 2:** 10 Countries with the highest number of HIV-associated maternal deaths, 2008, by model.

	Interagency		IHME	
Ranking	Country	^#^HIV-associated maternal deaths	Country	^#^HIV-associated maternal deaths
1	Nigeria	2472	Nigeria	10422
2	South Africa	1920	Malawi	4689
3	Zimbabwe	1574	Ethiopia	3971
4	Tanzania	1552	Tanzania	3941
5	Uganda	1512	India	3531
6	India	1264	Mozambique	3448
7	Mozambique	1217	Kenya	3006
8	Kenya	1100	Côte d'Ivoire	2871
9	Malawi	961	Uganda	2611
10	Ethiopia	948	Zambia	2403

**Table 3:** 10 Countries with the highest fraction of HIV-associated maternal deaths, 2008, by model.

	Interagency		IHME	
Ranking	Country	Fraction of HIV-associated maternal deaths	Country	Fraction of HIV-associated maternal deaths
1	Botswana	78%	Botswana	84%
2	Swaziland	75%	Swaziland	84%
3	Lesotho	59%	Lesotho	83%
4	Zimbabwe	53%	Zimbabwe	82%
5	Namibia	50%	South Africa	78%
6	South Africa	43%	Zambia	73%
7	Zambia	37%	Namibia	73%
8	Belize	35%	Malawi	69%
9	Malawi	32%	Mozambique	66%
10	Trinidad and Tobago	28%	Uganda	51%
